# Clinical Illustrations of the More Important Diseases of Bengal, with the Result of an Inquiry into Their Pathology and Treatment

**Published:** 1837-04

**Authors:** 


### Art. II.

1. *Clinical Illustrations of the more important Diseases of Bengal, with the result of an Inquiry into their Pathology and Treatment.* By William Twining, First Assistant Surgeon, General Hospital, Calcutta. 2*d Edition.—Calcutta and London*, 1835. Two volumes, pp. 481 and 438.

2. *A Treatise on the Functional and Structural Changes of the Liver in the Progress of Disease; and on the Agency of Hepatic Derangement in producing other Disorders; with numerous Cases exhibiting the Invasion, Symptoms, Progress, and Treatment of Hepatic Disease in India.* By W. E. E. Conwell, Surgeon of the Madras Establishment. —*London*, 1835. pp. 531.

3. *Transactions of the Medical and Physical Society of Calcutta.* Vols. VI. and VII.—*Calcutta and London*, 1833-1835. 8vo. pp. 509 and 497.

4. *The Indian Journal of Medical Science.* Edited by Frederick Corbyn, Esq.—Vol. III. *Calcutta*, 1836.

Various considerations induce us to look with complacency on the productions of the Indian medical press. In the first place, it is gratifying to observe that the zeal displayed by our European brethren to supply the deficiencies and correct the errors which disfigure the most ennobling of secular pursuits, finds its counterpart in the sultry regions of Hindostan, in spite of a climate singularly enervating to all, and of allurements to indolence and luxury which might be expected to seduce at least the young. Reflecting, moreover, that no inconsiderable proportion of our more juvenile readers may find in India a field for their talent and industry, we consider it our duty to arm them for the conflict they may have there to wage, by imparting to them the best information in our power regarding the works published in that country. We would remark, too, that our own seasons are not uniform, and that occasionally we have observed in those of unusual warmth deviations from the ordinary type of diseases, which rendered an acquaintance with the maladies prevailing in the vicinity of the tropics a matter of great practical value. The converse of this is likewise true: in the hill stations of Hindostan and the mountains bordering on Thibet, the type of disease approximates to the European, and we may thus in our every-day pursuits derive many a useful lesson from fellow-labourers so enlightened and zealous as those in India. When we consider, too, that there are in the Honorable Company's service alone eight hundred medical men who have received a good education dispersed over a territory comprising every variety of soil and climate, and countless inhabitants of extremely diversified physical constitution and manners; that there exists at Calcutta a medical society consisting of nearly four hundred members, and publishing Transactions which may vie in copiousness and originality with any in the western world; and that from the press of the same place there issues monthly a Journal of Medical Science, which, besides being freighted with "the wealth of Ormus and of Ind," takes acute cognizance of our own proceedings, the reader will acknowledge that we are sinking a shaft into a mine of almost boundless richness, calculated, (to use the expression of the excellent journal we have just named,) " to afford a source of heart-stirring interest to our brethren in Europe."

The " Illustrations" of the late Mr. Twining are characterized by that patience in research, philosophic modesty of induction, and sound judgment, which that able and estimable man carried into every pursuit in which he engaged; qualities which, we have no doubt, occasioned his being enjoined to undertake this work by the late Dr. Gibb and Mr. Ogilvy, the head of the medical department of the presidency of Bengal. Well has he discharged this flattering obligation! The prefatory essay on the Climate and Seasons of Bengal, and their influence in the production of disease, appears to us a model for such writings. In the moderate compass of fifty-four pages, we find information, precise without tediousness, on the climate and meteorology of the low lands of Bengal, of the mountain-stations of the same district, and that Montpellier of the eastern world, the Neelgherry mountains in the Mysore country. The rise and decline of epidemics according to the different seasons of the year; the influence of the climate on Europeans and on their children, and the question of tropical hygiène, find within the same space ample and judicious discussion. The author corrects a popular error regarding the supposed infrequency of urinary calculi in India.

"The Transactions of the Medical and Physical Society of Calcutta contain ample evidence of the frequency of stone in the bladder, both in Europeans and Asiatics. The splendid and numerous specimens in the Society's museum, furnished principally by Mr. Burnard of Benares, and Mr. Brett of Shajehanpore, and the history of the cases attached to those specimens, bear testimony to the frequency of the disease, as well as the skill and science with which those gentlemen perform one of the most dangerous operations in surgery. The successful operations of Messrs. W. Darby, W. Bell, and Dr. W. L. M'Gregor afford evidence of the frequency of the disease among the inhabitants in the vicinity of the great Himalayan mountains, and to the westward of the Indies, among the Seiks. In the lower provinces of Bengal, the disease is by no means rare. Mr. Egerton has recently operated on an European child, and on a native, in Calcutta, and numerous other cases might be mentioned. I have myself several times met with urinary calculi among Europeans, Indo-Britons, Hindoos, and Mahommedans." (*Illustrations*, p. 23-4.)

On the subject of *phthisis pulmonalis*, he confirms, from experience of the climate of Bengal, a remark we have often made in the south of Europe, that this disease, if it have attained the stage of softening, proceeds with greater rapidity in the warm than the cold or temperate regions of the globe. He allows that, in a less advanced stage, benefit is derived from a residence in India; his remark in this respect corresponding still with what is observed of the influence of the southern portions of our own continent on this disease. Asthma, he says, is as severe and intractable a disease in Bengal as in Europe.

The first disease illustrated by Mr. Twining is dysentery. This description corresponds in substance with that drawn by Pringle and Monro from cases witnessed in a different climate, and, with the exception of the phrase "pyrexia contagiosa," and with allowance for conciseness of expression, with that contained in Cullen's Nosology, thus confirming the view we have ever taken, that the difference between tropical dysentery and that of more temperate climates is one rather of degree and accidental complication, than of essential nature. Mr. Twining adopts the opinion now generally prevalent, and we believe generally true, that dysentery "is an inflammation of the mucous membrane of some part of the large intestines."

After a description of pathological appearances perfectly consistent with this opinion, but so minute and copious that we must leave its details to be perused in the work itself, the author proceeds to the important subject of treatment. We are happy to observe that, though this is so perfectly consistent with the pathology of the disease, that in its broader features it might be deemed, to use an expression of M. Louis, a corollary from it, yet those specialities, by which certain remedies are found to be applicable to the treatment of inflammation according to the structure and function of the organ it occupies, (specialities which only experience, direct or derivative, can teach,) are not neglected. This is exemplified in his abundant employment of ipecacuanha; a remedy, the great power of which over this disease is the subject of praise by writers contemporary with its first introduction into Europe. By the encomiums bestowed upon it by Magnatus, Piso, Ray, Johan de Lecat, Lemery, and Pomet, our enquiring and industrious author appears, from his reference to their works, to have been induced to give it that trial which in his hands has proved so successful.

The following is his account of the proceeding adopted in ordinary cases:

"*Treatment.* The cure of severe cases of acute dysentery in plethoric patients should be attempted, by the early, free, and repeated use of the lancet; with the aid of every other means by which we can subdue local inflammation and pyrexia. It will in general be requisite to bleed from the arm, two or three times, at the interval of eight or twelve hours, and to take as much blood each time as shall decidedly reduce, and permanently keep down any frequency and hardness of the pulse that may exist. And twelve or sixteen leeches should be applied soon after each general bleeding, to that part of the belly where pressure causes the greatest pain. In most cases it will be proper to continue this repeated abstraction of blood as long as pyrexia exists, or pressure on the belly gives pain, or there is any blood in the stools. A tepid bath should be used once a day, and the best time for it will be two hours after the leeches are removed. With this system of depletion, a dose of castor oil is given an hour after the first bloodletting; and when it has operated freely, the patient is made to take six grains of the powder of ipecacuanha, with four grains of extract of gentian, and five grains of blue pill, mixed and divided into three pills. These pills are repeated every night and morning; and twenty grains of powdered jalap, with forty grains of cream of tartar, are given daily at eleven o'clock in the forenoon. In ordinary cases of dysentery, I rarely deviate from these remedies, except by giving smaller doses of jalap and cream of tartar; and occasionally using calomel in place of the blue pill, and that is not very often done. In some patients, who have recovered from the more acute symptoms for many days, and have become emaciated, with dryness of the skin; I have omitted the purgative of compound jalap, and ordered a drachm of sulphur, mixed with half an ounce of mucilage, and one ounce of cinnamon water, to be taken in the morning early; the ipecacuanha, gentian, and blue pill being given at four and nine o'clock P.M. In such cases, the sulphur is a mild aperient, it has the property of acting on the skin, and I believe one of its effects in some stages of dysentery, after inflammation is subdued, is produced by its actual contact with the ulcerations of the intestines, inducing them to heal." (P. 69.)

After suggesting some familiar local remedies for tenesmus, and an injection of cold water for the painful affection of the bladder, with suppression of urine, which attends bad cases; and speaking of opium in terms which contrast strangely with the eulogies bestowed upon it by Drs. Armstrong, Cheyne, and others, whose experience of disease was confined to the British Islands; he gives the following dietetic precepts, which we think so rational, and so applicable to the dysentery of all climates, that we transfer them unmutilated to our pages.

"In all cases of acute dysentery, the greatest attention is requisite to the quantity as well as the quality of food and drink allowed; it being important to keep the colon as nearly as possible empty, while in an irritable state, and during the subsidence of inflammatory action, or the healing of ulcers. We must order the patient to be restricted to tea, barley-water, and thin sago, either of which may be given in the quantity of a coffee-cupful every six hours; and if the patient call urgently for drink in the intervals, a wine-glassful of weak infusion of chamomile flowers may be allowed every two hours: this, if used cold, is the best drink, and does not increase thirst. By attending strictly to this rule, it is probable that the food will be almost entirely absorbed in the stomach and upper portions of the small intestines. If the abstraction of blood be properly followed up, in a degree suitable to the inflammatory condition of the intestines; and the ipecacuanha, gentian, and blue pill, be given as directed; a restoration of alvine evacuations of healthy appearance, and removal of the uncomfortable sensations, take place so quickly, in most of the ordinary cases of dysentery which are treated at an early stage, that patients are with difficulty restrained from using an undue quantity and improper kind of food during the first days of convalescence, while the intestines are still weak, ready to be irritated by slight causes, and incapable of digesting any thing but the most bland food, in very moderate quantity. Errors in diet are the principal causes of tardy recovery, and of frequent relapses; therefore we must use the greatest caution, and only allow patients to resume their ordinary food very gradually, when convalescence is well advanced." (P. 73.)

Mr. Twining's views regarding both the pathology of dysentery, and the treatment it requires, are widely different, it will be observed, from the hyper-hepatic doctrines and the hyper-mercurial practice which our most popular tropical guides have hitherto but too successfully inculcated. He is candid, however, towards opinions which erred by ranking that as essential and primary which was but accidental and accessary. He does not altogether deny the affection of the liver, but reduces it to its due rank and importance.

"The usual causes of dysentery, being sudden alternations of temperature combined with an humid atmosphere, doubtless act by producing a degree of plethora and congestion of internal organs; and we can hardly doubt that the liver often partakes of this state. The most decisive means we possess, for relieving this condition of the liver, are the active depletion by bloodletting leeches, and purgatives recommended above; at the same time, that we use remedies to determine the circulation to the surface. These remedies may be sufficiently assisted by the small quantities of blue pill, or calomel, already advised, without producing salivation. Our earliest attention and most constant care, in the treatment of the acute dysentery of Bengal, must be directed to subdue the local inflammatory condition that exists. Many cases, if treated at the commencement of the disease, appear to be cured at once by bloodletting, leeches, and the tepid bath." (P. 75.)

Regarding mercury, we find none of those extreme opinions, marking an ill-balanced mind seeking to atone for one error by the commission of its opposite. It will be observed, that this mineral enters into the composition of the remedy which he employs as the great auxiliary to bloodletting in the disease. Against its abuse, however, he is a strenuous declaimer, censuring both the employment of large and repeated doses of calomel, given with the intention of producing salivation as speedily as possible, and of doses equally large followed by drastic purgatives. "When dark-coloured stools are observed," he says, "they are ascribed to disordered secretion of bile; and the patient, if dysenteric, is sentenced to undergo the discipline of large and repeated doses of calomel and drastic purgatives daily. If the stools are pale-coloured, calomel is still held in reverence as the best corrector of the disease. The pathology on which such practice rests is incomprehensible."

He asks the very reasonable questions, why dysentery, if ascribable to a disordered state of the bile, is confined to the large intestines; and why, if black and discoloured stools arise from the same cause, the contents of the small intestines should be of different shades of yellow and orange colour; whilst those of the great bowels alone are dark-grey, dark-brown, or black? In confirmation of the opinion implied in these queries, we may remark that, in cases which have terminated fatally when the patient was under the influence of calomel, (hydrocephalus presents itself as a disease in which we have witnessed such occurrences,) we have seen, on cadaveric inspection, the great intestines thickly lined with a substance of a dark colour, consisting of thin feculent matter and intestinal mucus; whilst the contents of the small intestines, (which, by hypothesis, should be the darker,) were of a pale orange or yellow colour. This dark-coloured matter constitutes the green or olive-coloured stools so often remarked, an appearance ascribed to the influence of calomel on the liver, but which we believe to be really due to its action on the intestinal lining; and that other irritants, tartarized antimony for example, or a diseased condition spontaneously occurring, will occasion the same phenomenon.

The author admits one exception to his denunciation of the "scruple-dose practice of India" in dysentery. This is when the disease is not above two or three days' duration, where the subject is robust, pyrexia considerable, and the calls to evacuate the bowels are very frequent, whilst only about half an ounce of reddish mucus is discharged; though, in such a case, besides a large bleeding, he associates with a scruple of calomel the same quantity of his favorite ipecacuanha. We cannot conceive a proceeding better calculated than this to produce an impression on an enteritic condition of considerable intensity; and we have adopted an analogous line of practice in similar cases in a climate more temperate than the Indian. Where dysentery is associated with scurvy, a disease of occasional occurrence in low and unhealthy situations in Bengal, he considers calomel, as might be supposed, peculiarly injurious. His remedies are, compound jalap-powder, ipecacuanha with extract of gentian, and a tolerably nutritious diet.

The disciples of the physiological or Broussaian school will feel surprised that Mr. Twining should, whilst the intestines are affected with an inflammatory disease, apply a stimulus to them such as compound jalap-powder, especially as he censures the employment of another stimulus,—large doses of calomel. The inconsistency may be reconciled by the fact that he has found the one stimulus injurious, the other beneficial; and that all sound therapeutics result from observation. That he has found laxatives useful, is no more than is observed in other forms of enteritic irritation, such as that connected with ulceration of the glands of Peyer, or constituting diffused muco-enteritis, in which the reasoning *à priori* of Broussais condemns them; but the experience of the profession proves the utility of their moderate employment, however this utility may be brought about; whether by the removal of irritating matter from the surface, increased exhalation, or on some other unascertained principle.

The author pays due attention to certain local affections complicating dysentery: of these, one of the most frequent and important is inflammation of the cæcum. This, when it occurs, generally takes place about ten or fourteen days after the commencement of the dysentery; and examination with the hand easily detects, in the right iliac fossa, a rounded, doughy, and inelastic tumour, which indeed is often visible to the eye. This tumefaction, depending on interstitial deposits of coagulable lymph between the coats of the intestine, or between the cæcum and iliacus muscle, may be discriminated from intumescence in the same region, arising from hardened fæces or flatus, by processes so obvious, that it would be superfluous to mention them. He declines entering into any disquisition regarding that inflammation of the cæcum and abscess in the iliac fossa, occurring independently of dysentery, which has been described so fully by Unger, Ferrall, Dupuytren, Husson, Dance, and others, and of which so ample an account was given in our second number.* He recommends for the treatment of the form of disease observed by himself, free local bleeding, with fomentation and poultice; an open blister on the part; a combination of Plummer's pill and extract of colocynth nightly, with a dose of compound powder of jalap in the morning; very spare diet, and the recumbent posture. In omitting general bloodletting from his list of remedies, he assumes that this has been performed for the primary disease, the dysentery.

Contraction and induration of the opposite extremity of the colon, its sigmoid flexure, frequently becomes the predominant affection in the latter stage of dysentery. The induration can be felt by pressure in the left iliac region; pain exists there, which in severe cases extends to the corresponding testicle; there is pyrexia, and the alvine evacuations vary according to the extent of the disease in the intestine, and to the presence or absence of affection of its internal coat, consisting in some cases of small figured fæces alternating with bloody slime, in others of merely sanguineous mucus. The remedies are in principle the same as those for inflammation of the cæcum. Mr. Twining very properly regards this affection as totally distinct from that stricture of the sigmoid flexure unfortunately not uncommon in this country, and which is of a carcinomatous nature. There is one very important ground of distinction, that, whilst the inflammatory disease, by timely and judicious treatment, admits of cure, the scirrhous scarcely receives palliation.

Our limits compel us merely to advert to much interesting matter on ulceration and destruction of the ileo-cæcal valve, and sloughing of the mucous membrane of the intestines; but we shall pause for an instant on the author's opinion regarding that "*vexata quæstio*," the mutual influence of dysentery and hepatitis. The facts of the case he thus states: the advanced stages of abscess of the liver are almost always attended with dysentery, and ulceration of the mucous membrane of the great

* See British and Foreign Medical Review. No. 2, p. 501.

intestines is found on the dissection of the subjects of these diseases; and, inversely, acute disease of the great intestines occasionally excites hepatic irritation, with tendency to suppuration; and sometimes deep-seated or central abscess of the liver takes place, in spite of the most decisive and accurate treatment.

Our author attempts an explanation of the latter fact only.

"This dangerous affection of the liver seems to arise from the abrupt cessation of copious secretions from the mucous membrane of the intestines, while some degree of the inflammatory condition is unsubdued; the decrease of discharge from the bowels giving rise to relative plethora of the mesenteric vessels, and consequent hepatic congestion and irritation. This consecutive hepatic affection is dangerous, because it takes place when the practitioner is generally relaxing in the diligence of his examinations, and remitting his remedies; as well as allowing more food, under the impression that his patient is cured." (Vol. I. p. 167-8.)

This partial explanation appears to us, and we express the sentimen with great respect for the memory of the writer, very unsatisfactory. So far from there being copious secretions in dysentery, the secretions are scanty, if we are to judge (and it is the sole means we possess of judging,) by the quantity expelled; whilst, estimating by the same test, secretion becomes more abundant as the patient approaches convalescence; the very period, says our author, when the hepatic inflammation arises. It should be remarked, besides, that one portion of the phenomena only is attempted to be explained; this hypothesis, and we can give it no more flattering appellation, leaving us in the dark as to the causation when the order is reversed,—the hepatic preceding the enteritic affection. We have no better explanation to offer, nor are we called upon to furnish one; but we would venture to suggest that, in whatsoever order the phenomena occur, whether from below upwards or inversely, the condition of the veins from the portæ to the branches of the mesenteric vessels should be diligently scrutinized.*

We cannot omit to notice the practice recommended by our author for that very intractable disease, chronic dysentery. So many modes of treatment have been found unavailing, that we are glad to notice one more for trial, and one which seems judicious.

"In the dysentery which is really chronic, there is seldom occasion for general bloodletting, as the system is commonly in a state quite the reverse of plethoric; but leeches are frequently of much service; and whenever indurations can be felt, unattended by morbid sensibility on pressure, or by any pyrexia, blisters are generally very important remedies. It is generally proper to begin the medical treatment of chronic dysentery with a moderate dose of compound powder of jalap, or of castor oil, for the purpose of carrying off the refuse of indigested food, or the remains of morbid secretions; after which, the patient should take ipecacuanha, extract of gentian, and blue pill, four grains each, every night; a drachm or half a drachm of sulphur every morning, mixed with cinnamon water, by means of thick mucilage or of powdered gum arabic; and a wine-glassful of the following mixture daily at noon.

* The author subsequently, after remarking that M. Gendrin's Histoire Anatomique des Inflammations affords instances of pus in the veins adjacent to ulcers of the intestines, asks this question: "May we consider the fact of pus being found in the veins of the intestines, as one link in the chain of events in those cases where abscess of the liver takes place at a late stage of bad cases of dysentery, attended by extensive ulceration of the intestines?"—Vide *note*, p. 232 *of Illustrations.*

R. Infus. Ipecacuanhæ ℥i.

—— Gentian. Comp.

Misturæ Camphoræ *aa* ℥v.

Tinct. Cardamom. Comp. 3ii.—misce.

"If there be any degree of morbid heat, with dryness of the skin, a mild purgative each morning, of compound powder of jalap, will be found most effectual; but if there be a dry and shrivelled skin, with coldness, the daily aperient of sulphur will usually answer best." (P. 179.)

He advises that the diet should be almost as limited in quantity as that directed for the acute disease, and that it should consist of sago, gruel, or other farinaceous food, with a little milk morning and evening, and one ounce of wine with the mid-day meal.

We refer the reader to the chapter on Diarrhœa for much interesting matter, but pass over it ourselves, in order that we may dwell longer on a subject very important to Indian practitioners, and requiring from those of Britain more correct appreciation than it uniformly obtains, *Disease of the Liver.*

It is difficult to convey a view at once clear and condensed of the author's elaborate description of the pathological appearances observed in the liver and its appendages; but the following enumeration of the heads to which the morbid conditions are referred will give the reader some general idea of the details:

1. Morbid changes in the gall-bladder; *α*, increased in size and distended with bile; *β*, decreased in size, false membrane covering it, and sometimes agglutinating it to neighbouring parts. 2. Enlargement of the liver, its colour dark, texture soft, bleeding freely when incised. 3. Abscesses of the liver, which may be, *α*, a large quantity of matter in a cavity, the contiguous parts not being much diseased, occurring in the course of fevers, acute dysentery, and in drunkards (acute central abscess); *β*, a small quantity of matter; the disorganization great; the parts on dissection being sloughy, like the advanced stage of a large carbuncle; occurring chiefly in the scorbutic; *γ*, a circumscribed abscess, the size of a small orange, in a cavity bounded by much coagulable lymph; *δ*, numerous small abscesses, the size of a filbert, dispersed through the substance of the liver. 4. Adhesion of the liver to the diaphragm, colon, or stomach, with more or less thickening of the peritoneal coat. 5. Black discoloration of a part of the liver, usually with some softening of its structure. 6. Tumours (probably glandular) varying from the size of a grain of barley to that of a bean, situated in the capsule of Glisson, causing pressure on the ducts, sometimes even their obliteration. 7. Pale, serous enlargement of the liver, the surface of the organ distinctly marked by the pressure of the cartilages of the ribs; occurring in chronic, leucophlegmatic disorders. 8. Liver of a pale slate-colour, with induration, and toughness of its texture. 9. Enlargement with paleness of colour, the texture soft and oily. 10. Induration and enlargement, the organ resembling in colour and texture boiled cow's udder. 11. Puckered depressions like cicatrices on the convex surface of the liver, with some induration from effusion of lymph beneath. 12. Biliary concretions in the gall-bladder. 13. Concretions like yellow soap, extending along the biliary canals; left lobe most frequently affected. 14. Enlargement (relaxation?) of the hepatic duct. 15.

Obliteration of the biliary ducts, observed only when the changes described in 9 and 10 had taken place. 16. Tubercles. 17. Hydatids.

There is one pathological condition of this organ unnoticed by the author, which has fallen under our observation in this country—diffused suppuration. The pus is not encysted, but constitutes an interstitial deposit throughout the structure of the liver, which is softened and broken down to the consistence of spleen, or even looser. The matter can be raised in abundance on a scalpel. Chronic inflammation with jaundice and febrile paroxysms, irregular in their period of recurrence, but each paroxysm resembling in character a fit of ague, has been the form of disease preceding this condition.

Regarding acute hepatitis, we are informed that its most unequivocal symptoms, and which are seldom deceptive, are a fixed pain in the region of the liver, which is not removed by purgatives, but is increased by pressure or by a full inspiration, a sense of oppression and fulness about the lower part of the chest, and across the epigastrium, at the same time that there is palpable enlargement of the liver.

There are two forms of the hepatic affection differing decidedly from each other, and neither of them quite identical with acute and superficial inflammation of the liver. These are inflammatory congestion with tendency to central abscess, and acute hepatitis with inflammation of the capsule of Glisson and adjacent parts.

The symptoms of the former affection are thus described:

"The enlargement of the liver, with tension at the epigastrium and across the hypochondria, is more evident in the early stages of this affection than in the acute peritoneal inflammation; there is also more oppression at the chest, attended with impeded respiration: but there is less of acute pain on taking a full inspiration, and less morbid sensibility on pressing over the liver, than in the acute superficial inflammation. One very common symptom of the incipient stage of this tendency to central abscess of the right lobe of the liver, is, a much greater degree of tension of the right rectus abdominis muscle than of the left; the muscle on the right side resisting pressure by a quick involuntary action, while the left rectus is lax, and other parts of the patient's belly are comparatively soft and elastic. I consider this one of the most undeviating symptoms of congestion, with incipient interstitial deposit into the texture of the liver, which commonly goes on to deep-seated abscess, unaccompanied by urgent symptoms of pain, or pyrexia. I have seen the left rectus muscle alone affected in this way, in patients who have afterwards died of abscess in the left lobe only. This symptom is of the more importance, as it takes place at an early period of the disease, when we can almost always effect a cure by due persistence in a proper system of treatment." (P. 243.)

The following is the account of the second form,—acute hepatitis with inflammation of the capsule of Glisson.

"The patients complain of pain at a circumscribed space about four inches above the navel, and to the right, on a line drawn from the umbilicus to the point of the right shoulder; and the disease is attended by the following circumstances. The attack sometimes commences suddenly after eating, and in that case the food is usually vomited, whereby a transient relief is experienced; the respite is but short, for the pain soon returns, and pressure over the part cannot be borne; a full inspiration increases the pain, and the patient is unable to stand erect, or to lie straight in bed; he rests with the body bent forward, and inclining to the right side; there is great anxiety, and the nights are passed without sleep; there is usually a sense of weariness and pain in the loins; tumefaction of the liver is seldom evident. In severe cases, the pain shoots back towards the lower angle of the scapula or up towards the shoulder; and is of the acute kind that is usually spoken of as a stitch or spasm, which prevents coughing or sighing. The bowels are usually costive at first, the urine is in general high-coloured, and jaundice sometimes takes place: there is a dry tongue, thirst, headach, and a frequent pulse, but not generally very high fever corresponding with the acute pain. In the latter stages of the disease, a distressing purging of black watery fluid takes place, and sometimes much blood is passed by stool. Severe cases, if not arrested by a very decisive and persevering treatment, will run their course in twenty or twenty-five days: during the last six or eight days of which time, the profuse discharge from the bowels usually attracts most attention; and the patient dies from irritative fever, produced by inflammation and congestion, which affect not only the liver, but the capsule of Glisson; and in some measure extend to the cellular structure round the duodenum, and at the root of the mesentery. It is not common for abscess in the liver to form, after the course of disease above described; though that is sometimes the case." (P. 244.)

The disease just described, and especially the form of it subsequently mentioned, differing from this only in being less acute in its symptoms and less rapid in its progress, and affections of intermediate degrees of intensity between the extremely acute and the chronic, constitute the most common type of hepatitis in this country. During any summer and autumn at once warm and moist, cases agreeing in all essential particulars with Mr. Twining's description fall under our observation in England. We have had other opportunities of testing this writer's descriptive skill; but familiarity with the diseases of this country alone would enable us to bear testimony to its accuracy.

The subject of Causes is handled with good sense and brevity. Those most efficient are stated to be a warm and humid atmosphere during the day, followed by cold nights. He admits that excess of wine and spirituous liquors, as well as of stimulating food, keeps many in India in an almost perpetual state of proclivity to inflammation and suppuration of the liver; yet he thinks habitual intemperance does not produce hepatitis so often as might be expected, but regards its influence on the result of the disease as very pernicious. The belief is expressed that hepatic abscess would less frequently follow delirium tremens, if this complaint, "when combined with febrile and inflammatory symptoms, were more commonly treated by antiphlogistic means." We concur in this sentiment, and earnestly recommend our professional brethren not to administer opium and stimulating remedies in a case merely because it would be not inappropriately designated "delirium tremens;" but to take the general condition of the system as their guide in its treatment; being convinced that under this term, and bearing external marks extremely similar, diseases of an essentially different nature are comprehended.

With Mr. Twining's simple and intelligible statement of causes, the ambitious enumeration of Mr. Conwell, diffused with characteristic prolixity and in endless divisions over twenty-seven pages of his work, forms a striking contrast. This is, indeed, a singular compound of facts and vague hypothesis, all propounded with an aphorismal solemnity which invests the most baseless speculation with the air of a self-evident and valuable proposition. We can gather from the introduction to this section, that he considers the form and distribution of the blood-vessels of the liver; the great caliber of the large vessels; the absence of valves; the abundance of capillaries; the proximity of these capillaries to the parent trunks, and what he terms "the triple capillary connexion interposed between the great sanguineous vessels, of which the centre is placed in the acini;" as tending to the production of congestion, or inflammation of the organ, and influencing either of these states to terminate in abscess.

These specialities of structure existing in every clime, we should have expected that inflammation, and even abscess of the liver, should be everywhere frequent diseases; but in our own country we find hepatitis rare in comparison of certain other phlogoses, pneumonia for example; and abscess of the liver *absolutely* rare. We shall leave Mr. Conwell to reconcile these facts with the peculiar proclivity to these affections with which the liver is endowed by its very structure. With regard to mere congestion giving rise to abscess, the enormous extent to which the former affection of the liver exists in certain diseases of the heart, without ever issuing in the formation of matter, remains likewise for his consideration. As to other causes, when we have disentangled his opinions from the speculative matters with which they are overlaid, we find them in accordance with those of Mr. Twining, excepting that he appears to attach more importance than this gentleman to intemperance as the direct source of hepatitis.

The treatment of the disease recommended by Mr. Twining bears the stamp of his practical good sense. He does not regard hepatitis as something special and peculiar, beyond the pale of those remedies which the experience of centuries has sanctioned in the treatment of inflammation, and refer us to mercury solely or principally for its cure; but insists strenuously on antiphlogistic measures, spare diet, and repose. An active purgative at the commencement, and general bloodletting repeated every six hours till the pain of the side and fulness of the epigastrium are relieved, form the basis of his treatment; and subsequently leeches are applied to the epigastrium every forenoon, beginning with twenty, and reducing the number to ten or six as the patient's strength becomes subdued, and the progressive subsidence of the disease may permit. After the purgative of the first day, it is advisable, he says, to give nightly ten grains of calomel with six of compound extract of colocynth, and four of extract of hyoscyamus, followed each morning by as much compound powder of jalap, or infusion of senna with salts, as shall produce four free stools in twenty-four hours. He is fully aware that it is one thing to relieve the more urgent symptoms of an inflammation, and another to cure it; he is therefore strenuous that the strict repose and the extremely abstemious diet, enjoined at the commencement of the disease, should not be relaxed on account of a mere remission of distress. If there is reduction of the patient's strength, tumefaction of the liver still remaining, his remedies are an open blister to the epigastrium, round the edge of which six or four leeches are daily applied; friction of the side every day, with half a drachm of camphorated mercurial ointment, till there is a commencement of salivation; and one or two doses of calomel or blue pill at intervals, to keep up the moderate mercurial action.

We cannot discover, in the forty-seven pages dedicated by Mr. Conwell to "a physiological review of the principal remedies used in the treatment of hepatic diseases," any that we are tempted to add to the practical precepts of Mr. Twining. We find here the same bewildering intermixture of vague hypothesis with ascertained facts which we have already had occasion to censure in other portions of this work. The remedies are arranged under eighteen heads, and the *modus agendi* of each, even the most familiar, such as bloodletting, is descanted upon as if it were perfectly new to the reader, and elementary works on materia medica and practice of medicine were not in existence. Something of this kind might be excused regarding croton-oil, which Mr. Conwell claims the credit of having first introduced into European practice; but even on this head we think he has somewhat abused his privilege. Some experiments performed with the oil by Magendie are reprinted from a work published by the author in Paris in 1823. They are not devoid of interest, and shew that this medicine acts by being absorbed and applied to the nervous system, and not directly on the muscular coat of the intestines.

There is a valuable paper by Mr. Geddes in the sixth volume of the Transactions on the subject of hepatic abscess. This is one of great interest in India, as is manifest from the following abstract of cases of hepatitis and liver abscess observed by Mr. Geddes in the Madras European regiment during the period of three years and ten months. Two hundred and twenty-eight cases were admitted under the denomination of hepatitis. In sixteen of these, abscess formed, and proved fatal. Besides which, twelve other cases of hepatic abscess terminated fatally in the same period, the patients having been admitted into hospital for the treatment of other diseases, principally fever and alvine fluxes. The whole number of deaths in the regiment during the period was 108, of which nineteen were owing to cholera: consequently more than one-fourth of the gross mortality was due to liver-abscess; and, if we deduct the cases of cholera, regarding them in some measure accidental, nearly one-third of the deaths arose from this cause.

Mr. Twining regards softening of the liver with sero-purulent infiltration as the preliminary or incipient stage of abscess; and this he has had an opportunity of observing in patients who have died from fever or dysentery, or from wounds and accidents during the progress of hepatic disease. This condition we have already adverted to as one which we have seen of itself fatal in this country. Inflammation and vascular engorgement he considers the precursors of this interstitial deposit.

The symptoms of abscess we cannot give more succinctly than in Mr. Twining's own words.

"In most cases the usual phenomena of hepatitis continue after suppuration has commenced, and frequently some other symptoms are superadded to those which previously existed. Rigor is not a general attendant on the formation of abscess in the liver, and that symptom was not observed in the majority of the cases which I have seen. When an extensive collection of matter has taken place in the liver, the pulse almost always rises above one hundred, and becomes softer and more readily accelerated by any exertion, or by change of posture from the recumbent to the erect position. In many patients, frequent cold perspirations are observed, attended with anxiety, debility, and a sunk countenance; in other cases profuse sweating occurs at night. Sometimes, in emaciated subjects, the pyrexia assumes the character usually observed in pulmonary hectic. During the rainy season in the low and damp stations of Bengal, proper remittent fever occasionally occurs in patients who are suffering from hepatitis, and if the liver be much enlarged, and not speedily reduced by active treatment, these cases are very apt to terminate in abscess. Mercury employed in the treatment of hepatitis does not very often cause ptyalism after suppuration has taken place; but if mercury be administered in such quantity as to produce salivation at an early stage of the disease, and sufficient depletion by bloodletting be neglected, the progress of inflammation, and the formation of abscess in the liver, are not certain of being arrested, and the existence of free ptyalism does not afford sufficient ground for assuring a patient that abscess of the liver cannot supervene. These are the more common constitutional symptoms of hepatic abscess; the local indications of existing suppuration are very often an increased tumefaction of the liver, the patient suffers more from oppression at the chest and anxiety; and in those cases where the liver is so large as to protrude beyond the margins of the ribs, and the abscess is seated anteriorly, a more or less circumscribed projection takes place as the disease advances. If the abscess be situated at the concave surface near the stomach, vomiting often occurs; but this symptom is not a very unfrequent attendant on abscess of the liver seated remotely from the stomach." (P. 295.)

Central abscess often forms without so much pain as to excite the alarm of the patient, and these cases, as the author justly observes, prove the skill of the practitioner more than the sudden attacks of acute inflammation with severe pain. The following symptoms are mentioned as the indications of this obscure form of affection:

"The continuance of slow fever, with protracted diarrhœa, and tension of the recti-abdominis muscles, should make us very vigilant in our observations and careful in the treatment which we order, as we know that liver-abscess frequently runs its course in Bengal without active pyrexia, and with but little pain in the part affected; and sometimes without any palpable enlargement of the liver. Those persons in whom abscess in the liver proceeds in this way, lose their health in a very gradual manner, becoming emaciated, and having returns of fever for a few days, once in two or three weeks; they then suffer from slight pyrexia almost every day, attended with more or less of diarrhœa, and followed after some weeks by hectic fever and quick pulse. The muscles of the abdomen, in these cases, are usually somewhat tense, especially the right rectus; the belly is frequently not tumid, and a careful examination detects only a trivial enlargement of the liver." (P. 297.)

We have seen in this country cases of abscess of the liver bearing a general agreement with the foregoing description. In certain cases there was total absence of pain in the hepatic region; and there were exacerbations of a constantly existing degree of pyrexia, recurring at intervals varying from a week to a fortnight, or longer. These exacerbations were, however, (in most of the cases we have observed,) ushered in by a strong rigor, which, conjointly with the general course of the subsequent phenomena, imparted to them much of the character of an ordinary fit of ague. If jaundice formed part of the symptoms of the disease, the yellowness became more intense during the exacerbation, and remained so for a day or two. Mr. Geddes, in the excellent paper we have already quoted, mentions rigors, investing the pyrexia with the character of a paroxysmal fever, among the symptoms of the disease. From the same authority we derive ample confirmation of Mr. Twining's statement regarding the extreme uncertainty and obscurity of the indications of hepatic abscess. It appears that, of the twenty-eight cases which he reports, pain existed in only thirteen, dysentery in ten, and pyrexia in but five cases.

The last-mentioned circumstance is so startling, that, but for the strong evidence of accuracy furnished by the general tenor of his very elaborate article, we might have suspected some error. He informs us, moreover, that, of the twenty-eight cases, sixteen only were designated liver or hepatic disease; five being considered dysentery; two continued, and one intermittent, fever; two abdominal inflammation; one chronic diarrhœa, and one debility. Whilst he regards pain in the region of the liver or its vicinity, or in the right shoulder; dysentery, or diarrhœa; fever, or fever and pain conjointly, as the strongest diagnostic marks of this affection, he seems to consider pain and dysentery as mutually excluding each other; remarking that, where the dysenteric symptoms are severe, there is little pain; and inversely, where the pain is urgent, there is little affection of the bowels. We see in these statements of Mr. Geddes obvious reasons for concluding that the existence of hepatic abscess is not to be inferred from this or that pathognomic sign; but, in a great majority of instances, only to be strongly suspected from an assemblage of circumstances and symptoms.

Mr. Twining, aware how hopeless a case is presented by abscess of the liver when fully formed, is urgent that no pains should be spared to obviate so melancholy a consummation. Impressed with a belief that the interstitial deposit to which we have more than once adverted, and which he regards as the preliminary stage of abscess, admits of absorption; and considering the chances of recovery from such a process as infinitely greater than from the bursting of an abscess even in the most favorable direction, he advises, if the patient's strength is unsubdued, and any inflammatory symptoms remain, that we should, notwithstanding any indications of effusion, persevere in the antiphlogistic measures recommended for the primary affection. These reasonable views of the author receive confirmation from several of the cases detailed by him.

Under circumstances differing from the foregoing, when the abscess is fully formed, the patient emaciated and weak, and suffering from hectic and colliquative sweats, we are recommended to support his strength with mild and nutritious diet, and to endeavour to inspire cheerfulness of mind. Many patients die before the abscess bursts. In other cases it discharges itself either into some part of the intestinal canal, or, through the diaphragm and lower part of the lung, into the bronchial tubes; or externally through the parietes of the abdomen, or at the lower part of the side of the chest. A proportion, but only a small proportion, of such cases recover under a careful and discriminating medical treatment. This Mr. Twining very properly considers as consisting in a mild diet, given in sufficient quantity to prevent the patient's strength from sinking; whilst, on the one hand, any endeavours to produce plethora, and, on the other, debilitating measures, such as purgatives, mercurials, and leeches, are avoided. Where fluctuation can be ascertained, or where there is an evident pointing externally, abscesses should be opened, he thinks, more frequently and at an earlier period than is now practised. We regret to add, that he witnessed but one case of recovery. In this, the incision—and this method he prefers to caustic,—was performed at the epigastrium.

No writer conveys more distinct ideas than Mr. Twining on a subject regarding which the public has borne much written and, we fear, not a little of practised quackery,—chronic diseases of the liver. The majority of these, he thinks, differ from the acute only in degree and duration, and require for their treatment the same measures, modified, in their force and the period during which they are applied, by the circumstances of the complaint. Illustrative cases are given. The examples of chronic inflammation of Glisson's capsule, the gall-bladder and adjacent part of the liver, with disorder of the duodenum, will be read with much interest. The seat of the tumefaction and pain, just below the centre of a line drawn from the umbilicus to the right nipple; the increase of this pain after taking food; the slow fever and emaciation; the diarrhœa, with scanty and dark-coloured evacuations, and the result of the practice employed, local bleeding, low diet, and moderate mercurial purgatives, confirm the accuracy of the view taken by the author, of these affections.

Two forms of chronic hepatic disease, besides the inflammatory, are described. 1st. The red disorganization, with slight enlargement, induration and irregularity of the surface of the liver. This morbid condition arises from hypertrophy of the red substance of the liver, and some obstruction to the venous circulation. Red blood of an unhealthy description abounds in these patients. They suffer much from various constitutional ailments, and are distressed by disorders of the digestive organs. They generally die from extreme emaciation, which takes place rather suddenly, and which is attended with a troublesome cough. 2d. The pale degeneration of the liver, which is attended with some enlargement. It depends on hypertrophy of the yellowish-white substance of the organ, and there is more or less induration. It occurs most frequently in persons of pale complexion, who have been long resident in India; the patients are bloated, fat, and dropsical; they are sometimes slightly jaundiced.

On the subject of *Jaundice*, we find in Mr. Twining's work much interesting matter, pathological and therapeutical. The pain, which generally exists in this disease, and which is increased by pressure, is so situated with reference to a line drawn from the umbilicus to the right nipple as might lead to a belief that inflammation of the portion of the liver adjacent to Glisson's capsule was the origin of the affection. Reflecting on the absence of jaundice in many cases of extensive inflammation of the liver, and even suppuration involving large portions of the organ, the author was induced to suspect an error in his pathological views, and to pursue the investigation. This led to his discovering, besides infiltration of coagulable lymph into the cellular tissue of the capsule of Glisson, an enlargement of two lymphatic glands situated within this capsule, one adjoining the cystic duct at a little distance from its origin, the other lying at the side of the ductus communis. The former gland, if enlarged, pressing only on the cystic duct, it cannot be supposed that jaundice could arise from its tumefaction. Not only pressure of the common duct, but its actual obliteration, has been observed from swelling and induration of the other gland; and we need scarcely remark that, in the former circumstance, if to any considerable extent, and unequivocally in the latter, we have an ample efficient cause of jaundice. These important views of Mr. Twining are confirmed by reference to the normal condition and situation of these glands; by the pathological researches of Mr. A. K. Lindesey and Mr. D. Stewart, in the seventh volume of the Calcutta Transactions; and by a case published in the Lancet for February 8th, 1834, by Mr. G. Jones. The high authority of Andral is also in favour of the operation of this mechanical cause of jaundice. He says, speaking of the morbid phenomena produced by enlargement of the lymphatic ganglia within the abdomen, " when those diseased ganglia are accumulated round the hepatic duct, they compress its parietes, the bile no longer flows into the duodenum, and jaundice ensues."*

It is but justice to Mr. Twining to mention that his discovery of the diseased condition of these glands preceded his acquaintance with M. Andral's work; and was anterior to the observations of the same fact in other quarters.

Where the liver is enlarged, and the general symptoms of hepatitis are associated with jaundice, the bile at the same time passing into the intestine, there can be no doubt of the propriety of employing, as already advised for the primary disease, bleeding, general and local, calomel, and active purgatives. When no enlargement of the liver exists, but there is circumscribed pain in the region of the gall-bladder and ducts coming on gradually, and white or clay-coloured stools, the antiphlogistic part of the treatment is adopted; but it is suggested that mercury should be entirely omitted. Agreeing with the author in the expediency of venesection, leeches, purgatives, and the warm bath, as most efficient remedies of jaundice, whether it occur in India or elsewhere, we beg to dissent from the negative precept regarding mercury. He seems to be influenced in his opinion by the remark of Dr. Cheyne, that, in large establishments for the cure of venereal disease, jaundice often appears during courses of mercury. Admitting the correctness of the remark, and stating the corroborating circumstance that, under severe ptyalism, the alvine discharges are almost always clay-coloured or white, we must still be allowed to regard the *moderate* employment of mercury as an important auxiliary in jaundice to the depletory means recommended. The biliary duct is obstructed, says Mr. Twining, and to give mercury with a view of exciting the secretion of bile would be as unreasonable as to administer diuretics to a man with a distended bladder, when we knew he had impervious stricture of the urethra. We reply that we would not exhibit mercury with the view of increasing biliary secretion, but with that of aiding to surmount the inflammation on which the author, equally with ourselves, believes the obstruction to the flow of bile into the intestine to depend. Because mercury has been grossly abused in India and other parts of the world, we cannot see the reasonableness of foregoing the benefit derivable from its use in inflammation. On referring to Mr. Conwell, we find that, whilst he makes no mention of bleeding, general or local, he advises the prudent employment of mercurial and other purgatives, diluents, sudorifics, and the warm bath. He appears to us, however, by omitting the most important of remedial measures, to be securing to his patients a tedious disease and a protracted convalescence; but his very vague and speculative views of the pathology of the disease are not calculated to suggest a very decisive practice.

The chapter in the Clinical Illustrations, of which diseases of the Spleen are the subject, is not inferior in interest to any in that valuable work. Splenitis, or acute inflammation of the peritoneal coat of the organ, the author tells us, is a rare disease in any country; an opinion in which we concur. But there are diseases of the spleen, consisting principally in its enlargement, softening, and vascular engorgement, which are frequent in India and elsewhere, and very important with

* Andral's Pathological Anatomy, by Drs. Townsend and West. Vol. ii., p. 457.

reference to the constitutional condition in which they originate, or with which at least they are associated. This condition is marked by the early accession of general debility, paleness, and a deficiency of blood in the capillary vessels, most remarkable in the pale and bloodless aspect of the conjunctiva, hectic blueness or pearl-colour of the sclerotica, and chlorotic discoloration of the face, tongue, and gums.

The symptoms of the disease vary according to age and sex, though all seem mainly referrible to anæmia and extreme debility. Children display, as might be supposed, less power of resistance under it than adults, and, when attacked, if proper medical treatment be omitted, they speedily fall into a state of deplorable marasmus. They become languid and weakly, and their breath and the exhalations from their bodies have a nauseous and sickly odour. Females under the same condition, besides the general indications of anæmia and debility, generally suffer from amenorrhœa. In the few cases in which menstruation is not obstructed in such subjects, there is a favorable prospect of recovery.

Various indications of an attenuated state of the blood exist. It coagulates imperfectly, or the cruor is black and soft, and its surface does not assume, on exposure to the air, the usual florid appearance. There is no buffy coat, unless pyrexia or acute pain of the left side be present. Trifling causes give rise to ulcers, which, from a deficiency of constitutional energy, show a disposition to spread. This connexion between disease of the spleen and the ulcerative process was long ago pointed out by Morgagni, and even by Celsus;* but we feel not the less obliged to Mr. Twining for recalling our attention to it. There is a tendency to hemorrhage from slight injuries, and profuse discharges of blood occasionally take place from the stomach, which, the author remarks, may flow from vessels directly communicating with the splenic vein; as, when the spleen is swollen, great relief is thereby afforded to it. It may be worth remarking, that the fact of the detumescence of the organ after such sanguineous discharges, and the opinion that they proceed from vessels directly communicating with those of the spleen, are noticed by the authority we have just quoted and several of the older writers.†

Patients labouring under this disease are affected with a short and imperfect respiration, their appearance evincing that decarbonization of the blood is inefficiently accomplished; and any attempt to take active exercise excites panting and distress in the chest. The appetite is impaired, the digestion difficult, and the food appears to be imperfectly assimilated; in a few instances there is morbid craving for nourishment. There is despondency and depression of spirits, inactivity of body, and torpor of mind, with great muscular debility; and this latter symptom is remarkable, although the patients be not much emaciated. The disease, when fatal, is so through the intervention of dysentery or ascites.

Intermittent and remittent fevers give rise to many of the cases of tumefaction of the spleen which occur in Bengal; this organ being very obnoxious, as is familiarly known, to the influence of these febrile affections, as M. Louis has shown it to be to that of the typhus of Europe. But, independently of any marked causation of this kind, its enlargement may

* Morgagni, lib. iii. Epist. 36, cap. 17.—"Ulcera aut omnino non sanescunt, aut certè cicatricem vix recipiunt." Celsus, lib. iv. cap. ix.

† Morgagni, lib. iii. Epist. 36. cap. 12.

arise as an idiopathic disease in children, and persons of a delicate and feeble constitution, under the combined influence of a damp climate, variable temperature, deficient exercise, unsuitable clothing, and insufficient nourishment. The depressing passions constitute, in some persons, a direct or co-operating cause of the affection.

After remarking, that the assemblage of constitutional symptoms described, and to which he gives the very appropriate name of *splenic cachexia*, may exist where neither enlargement nor morbid sensibility of the organ is very palpable, the author adds, that the disorders most closely allied to it are "chlorosis, scorbutus, and some species of anæmia." We certainly consider the analogy to chlorosis presented by certain degrees of this disease as very striking indeed, and participate in the surprise expressed by the author that it has not been generally noticed. Besides the indications of an anæmious condition which in chlorosis are so manifest, the fixed pain in the lower part of the left side, which is uniformly complained of, we have in some cases traced, by examination, to an enlargement of the spleen. The degree of swelling required to protrude the organ beyond the margin of the ribs being very considerable, this physical sign cannot, in the majority of instances, be obtained; but the seat of the pain, and reasoning by exclusion, lead to the belief that it arises from the spleen. The argument to be drawn from the successful application of an identical treatment to both diseases is very powerful.

The treatment recommended by Mr. Twining for vascular engorgement of the spleen consists in the administration of purgatives, combined with bitters and the sulphate of iron. The formula usually prescribed by him contains jalap, rhubarb, calumba, ginger, supertartrate of potash, and sulphate of iron. The close analogy of these with the remedies ordinarily employed for chlorosis in this country, aloetic purgatives and iron, will at once strike the reader. The coincidence with the remedies recommended by Celsus for spleen-disease, is likewise very curious: "Potui sero jejuno dari solet absinthium incoctum; et post cibum aqua à ferrario fabro in quâ candens ferrum subinde extinctum sit; *hæc enim vel præcipué lienem coercet*."* The idea intended to be conveyed by the words of this writer distinguished by italics appears to influence the practice of the natives of India of the present day, whose remedies are, in substance, the same as those already mentioned. They think that the application of so strong an astringent as sulphate of iron to the stomach, in the immediate vicinity of the spleen, tends to contract the latter organ; for, when this medicine is administered, the patient is directed to lie on the left side, that it may flow to the part of the stomach in contact with the spleen. Mr. Twining states, that, in some young subjects, placed under his care in a very advanced state of the malady, who fell victims to spleen-disease, and to whom he had given sulphate of iron in comparatively large doses, he found the stomach quite white and exceedingly contracted, "more resembling a man's thumb than a young child's stomach."

In certain obstinate and tedious cases, the author resorts to the remedies of the natives, and speaks favorably of their effects. Besides the internal medicines, consisting of purgatives, bitters, stimulants, and some preparation of iron, or the sulphate of copper, they introduce

* Lib. iv. cap. 9.

needles into the swollen organ, or apply the actual cautery to the integument corresponding to it. Of the beneficial effect of the former proceeding, the author speaks from his own experience; and of that of the latter, from the cures he has seen effected by it on Hindoos by native practitioners who had employed it. Not the least interesting portions of the work are such notices of native practices with which it is interspersed, strikingly resembling, be it remarked, those of the Greek, Roman, and Arabian physicians; from which last source they are probably directly derived.

A long section of Mr. Twining's work is dedicated to the consideration of the Effects of Mercury on enlarged Spleen. From the predominant anæmia and asthenia with which such enlargement is associated, most practitioners would augur unfavorably of the employment of so debilitating an agent as mercury; and the experience of the author justifies such apprehensions. After detailing many valuable cases, and bestowing much sensible and candid criticism on the opinions and practice of many writers, —Indian, British, and foreign,—he arrives at the conclusion that we should not give mercury where enlargement of the spleen exists, or the constitutional symptoms of splenic cachexia are strongly marked.

A chapter of nearly two hundred pages is dedicated by Mr. Twining to *Cholera.* We need not say that it is sensibly written, but, having no claim to novelty of opinion or practice, (the only qualities which can give interest to a treatise on this subject in England,) we shall take the liberty of passing it over without further notice.

The subject of *Fever* occupies, as might be supposed, a large space in the volumes before us. In some general remarks, Mr. Twining argues against the doctrine which attributes all fevers to local inflammation. His principal argument is the same as one which M. Louis has employed, and is derived from the early stage of fever, when there exist abundant indications of constitutional affection, but none whatsoever of local inflammation. Fully convinced as we are of the perfect independence of the state of fever, we yet feel some doubt regarding the force of this argument; conceiving that we have observed, before the occurrence of decided phlogosis, a considerable degree of indisposition preceding (if the expression be allowable,) the localizing of the disease. Whatever may be our doubt of the argument, we feel none, however, of the doctrine it is adduced to support; and we are glad to observe able men in all quarters of the world stepping forward to advocate this truth, relating, as it does, to a question of practical pathology, not one of idle speculation.

The division of fevers in the Clinical Illustrations is very simple,—viz. into intermittent; the common continued fever of the hot season; the remittent fever of the rains; and the insidious congestive fever of the cold season. The yellow fever is hardly to be accounted an endemic of Bengal, although every year patients are met with in whom a yellow suffusion of the skin occurs in fevers of considerable severity. From Mr. Conwell's statement, it appears, however, that in the Madras presidency, particularly at Penang, yellow fever (to which he gives the name of *congestive nervo-bilious fever*,) is a frequent disease. To prove its identity with the disease of the western hemisphere, he quotes nearly forty pages from the writings of Hillary, Hunter, Gregory, Chalmers,

Chisholm, Rouppe, &c.; and, though we admit that he has proved his point, we think he might have compressed his evidence into smaller compass.

The frequency and obstinacy of the visceral complications with which intermittents are associated in Bengal, constitute their most important characteristic. These complications may exist in the meninges or the brain itself; in the liver; in the spleen; in the mesenteric glands and cellular structure at the root of the mesocolon and mesentery; and in the lungs. Though not uniformly existing early in the disease, they form in the course of it; and that practitioner will be most successful in his treatment who displays the quickest perception in detecting their existence, and the greatest skill in removing them. The prevalence of these visceral complications renders the intermittents of Bengal less amenable to quinine and other antiperiodic remedies, than those of some other climates. The following is Mr. Twining's plan of treatment: —He purges the patients freely at the commencement of the disease, administering his purgatives at the conclusion of the hot stage. Should the different stages of the paroxysm be severe, and attended with distressing symptoms affecting either the head, chest, or abdomen, he strongly advocates the necessity of having recourse to venesection, according to the plan of Dr. Mackintosh, noticed in our last Number. Mr. Twining recommends that the medical man should be near his patient when the cold stage comes on, in order that he may be prepared to draw blood, from a large orifice, at the commencement of the rigor, or when coldness and shivering are completely established. The quantity drawn must be regulated by the effect on the rigor, and in some degree by the size and condition of the patient as to plethora; but twenty ounces are considered the greatest amount to be taken from an European, and twelve from a native. The blood is to be drawn from the patient in a recumbent posture; he is to lie quiet for an hour after the bleeding; he should take a little warm liquid, such as tea, and ought not to be too much covered with bedclothes. If these means be adopted, there will seldom be either a hot or a sweating stage; and, if purging have been freely employed, there will probably not be a return of the paroxysm. Besides this general bleeding, leeches should be applied to the seat of the local affection. Many cases, however, occur in Bengal, in which sulphate of quinine, liquor arsenicalis, or some other form of antiperiodic medicine, is required to prevent a recurrence of the paroxysms. The author's remarks on the cases to which these medicines are respectively applicable are extremely judicious; but, there being no novelty in them, we shall abstain from transferring them to our pages. He mentions some remedies very familiar in India, with which we are but little, if at all, acquainted. Among these is the kernel of the Kutkuleja nut, the seed of the Cæsalpinia Bonducella. Ten grains of this substance, with four of long pepper and two grains of assafœtida, in pills, twice a day, he has found a very efficacious prescription for natives, in whom the disease has existed long and the feet are slightly swollen, without visceral disease. The Chiretta, (Gentiana chiretta,) of the tonic powers of which he thinks very favorably in many diseases, is likewise one of his remedies for ague, either alone in a strong decoction, or in the form of a weak infusion, as a vehicle for *liquor arsenicalis.* This last medicine he rerecommends in doses of six drops for an European, and four for a native, repeated every two hours; observing in this case the general proportion in which he advises medicines to be given to these two classes of men respectively.

The *Continued Fever* of the hot season is a disease with which every individual accustomed to practise among Europeans in the warmer latitudes is perfectly familiar. It is the purest example possible of the *Synocha* of our nosologies. The general system is in a state of high excitement; the pulse is frequent and full; the skin is hot and dry; the thirst is ardent; there is great headach; and the prostration of strength is sudden, and often considerable. Local inflammations manifest themselves early, their seat being the brain, the stomach, the cellular structure round the duodenum and at the root of the mesentery, and the liver. Where the brain is principally affected, there is indifference approaching to stupor; when the stomach mainly suffers, nausea and vomiting occur, and the tongue is either loaded with yellowish mucus or brown fur, or it is dry, pointed, and red at the tip. In the event of a fatal termination, the morbid appearances correspond with the symptoms during life, consisting in sanguineous congestions in the organs mentioned; and, if the disease have lasted for some days in a violent degree, sero-albuminous effusions are found on their surface. This disease frequently attacks plethoric individuals recently arrived in India, and its ordinary causes are insolation, immoderate exercise in the hot season, bathing when heated, costive bowels, and improper indulgence in wine or spirits.

The treatment required in so plain a case is very manifest. It consists in the early application of a depletory and antiphlogistic practice, the patient being bled to the amount of a pound or a pound and a half at the commencement of the disease, and smaller quantities of blood being subsequently drawn according to the resistance of the symptoms, whilst leeches are applied to the vicinity of the organs affected. A large dose of calomel and colocynth should be given after the bleeding, and, six hours having elapsed, a purge of salts and senna ought to be administered till it acts freely. Mr. Twining regards tartar emetic, given in doses of the sixteenth of a grain in half an ounce of fluid every hour, after the patient has been freely purged, as next in efficacy to bleeding.

"Remittent fevers," Mr. Twining remarks, "are characterized by a diurnal exacerbation and remission of the pyrexia; the paroxysms not coming on with rigor, (though in some cases the commencement of the disease is ushered in by shivering;) the hot stage variable in intensity and duration; followed by perspiration which is often profuse, and occasionally attended with sudden and extreme prostration of strength. The intervals between the paroxysms are often marked by entire cessation of pyrexia. In some of the worst cases of this disease, the phenomena which occur during a paroxysm observe no regular order of succession." (Vol. ii. p. 287, 288.) He subsequently acknowledges that no concise definition can comprise all the varieties of remittent, modified as the disease is by individual peculiarities, circumstances appertaining to the general atmospheric constitution, those existing in certain localities, and by the nature and extent of the congestions with which it is uniformly associated. In its slighter varieties, it differs but little from the irregular intermittent connected with functional disorder of the digestive organs; while the more malignant types, occurring during unhealthy seasons, resemble the *pernicious* intermittents of the fens of the south of Europe, which are characterized by the symptoms of extreme congestion of blood in one or more vital organs, by the early accession of debility, oppressed respiration, a small and weak pulse, anxiety, and the predominance of cold perspirations. Probably the only characteristic of the whole tribe that can be regarded as strictly pathognomic is the marked exacerbation; an exacerbation much more considerable than any occurring in the course of a fever called continued, which takes place once or twice in the twenty-four hours. This arises either at eleven A.M. or at nine P.M., or there may be two paroxysms occurring nearly at the periods mentioned. The local congestions which attend this disease often pass rapidly into inflammation, attended with much interstitial effusion. They are seated in the stomach, intestines, cellular structure about the duodenum and at the root of the mesocolon, more especially where it passes across the spine. They likewise often occupy the spleen, liver, brain, or lungs; but there is a great diversity in the relative degree in which these or any organs are affected.

The rapidity with which changes take place both in the disease and the powers of the patient's constitution, renders the treatment requisite in Remittent Fevers at once decisive and delicate. The portion of the Illustrations in which this is detailed is of a character to convey a strong impression of the power of observation and the practical skill of the writer. The employment of that important remedy, bloodletting, should, if possible, take place soon in the disease; the most appropriate periods being the early stage of the first and second paroxysm: later, it is a more doubtful remedy, and demands much caution. Should there be high arterial action, or distinct symptoms of local inflammation, it may be employed later, even on the eighth or ninth day; but, when resorted to at such a period, the patient should be carefully watched; and every accessory remedy, such as quinine, food, and wine, must be administered in proper time. Bleeding to syncope is accounted extremely injudicious: whatsoever induces fainting being found to have a prejudicial effect on the condition of the patient. Local bleeding by leeches to the head or epigastrium, as the symptoms of congestion may indicate, requires less caution. The more powerful purgatives are required in the earlier stages of the disease; but, in a protracted disorder, even in its inflammatory forms, and at all periods in the weak and delicate, and in a low type of the disease, their employment requires care, and we must often substitute for them mild aperients and enemata. Mr. Twining does not consider the frequent repetition of large doses of calomel necessary in any case of the disease, seldom going beyond two doses of twenty grains, and one or two of ten grains in the severest examples. He regards the benefits of salivation as problematical, though he thinks that he has seen in some cases, where there was evidence of effusion having taken place in the brain, a favorable change sometimes immediately precede, and at others follow, this effect of mercury.

One of the most material points of his practice is the exhibition of the sulphate of quinine, during the remission, to prevent the recurrence of the paroxysms. His directions for the management of this powerful agent seem to us extremely suitable to the delicate circumstances in which it is employed. He thinks less favorably of its use where the cerebral affection seems considerable; but, when other local inflammatory disorders exist, a few small doses given in solution during the apyrexia, frequently alter the character of the malady, and enable the medical attendant to subdue the topical complication with greater ease. When summoned late to a protracted and bad case, he sometimes administers it without previous purging, thinking justly that to arrest a paroxysm is to save the patient's life. The most suitable period for giving it is during the remission, when the patient is perspiring, and probably languid from the preceding excitement. The dose generally employed is four grains, repeated so that about three portions may be taken in the apyretic period. He sometimes associates it with the purgatives employed.

*The Congestive Fever of the cold season* is an insidious form of disease, characterized, at the commencement, by slight and obscure symptoms. So slight, indeed, are they, amounting to little more than a feeling of lassitude, occasional transient pains in the joints, and inaptitude for intellectual exertion, that the patient continues, in spite of them, his daily business and nightly amusements, for a period varying from three or four days to a fortnight. During this time he probably aggravates his indisposition by taking strong soup, and more than an usual quantity of wine, to relieve the debility under which he considers himself to be suffering. When medical assistance is summoned, the pulse is found to be soft, frequent, and weak; the wrist tremulous; the tongue but little disordered; and there are anxiety and a general sense of weariness. Pain in the forehead exists; the eyes are weak; the bowels are generally costive; and there are fulness and tension across the epigastrium and in the hypochondria. Unless prompt and efficient measures are adopted, there is a slow increase of pyrexia, and nightly delirium supervenes, with drowsiness and stupor during the day; the eyes become red; the tongue much loaded, brown, and dry; the urine high coloured, and sometimes a yellow suffusion of the skin and eyes takes place. An occasional symptom is great soreness of the whole body, particularly perceptible along the course of the absorbents and in the glands, where they are subjected to pressure. On the occurrence of this fever a short time prior to the period of menstruation, the practitioner sometimes delays the use of active remedies, particularly bloodletting, on the principle of not interfering with the course of nature, and for fear of arresting the monthly discharge. The immediate effect of such indecision is an increase of the febrile state; its more remote one the author considers to be that chronic thickening of the *os* and *cervix uteri*, on which dysmenorrhœa frequently depends.* If the fever be slight, and in a delicate person, the medical man may trust its cure to rest, diluents, and aperients; but, under other circumstances, venesection ought to be employed, and even repeated, as in a similar attack not coinciding with the menstrual period.

Dissection does not discover much change of structure different from that found in remittent fever, excepting that superficial ulcerations of the mucous membrane of the small intestines have been found in a few rare instances. Should future observations prove that these ulcers exist

* Professor Autenrieth has observed a similar effect from the occurrence of scarlatina, under parallel circumstances.—*Annals of Med. and Surgery,* vol. ii., p. 292.—Rev.

generally in fatal cases, Mr. Twining would consider that a peculiarity of the disease is ascertained, which, conjointly with its character and the season at which it occurs, might establish a resemblance to some modifications of European typhus. Admitting the general resemblance in other points, we cannot suppose the superficial ulcerations mentioned by the author to bear much similarity to the extensive affection of the glands of Peyer described by the French and other pathologists, or they could not have escaped so diligent an investigator, in the more frequent order of cases in which they were not observed. It appears to us, therefore, either that they must materially differ from the corresponding affection in European typhus, or that they must be rare in the Indian malady, instead of being *generally* found, as in the former disease.

At the close of his work, Mr. Twining has an interesting chapter on the constitutions of the natives of India. The subject is so extensive, the population comprising many races, that the treatise cannot be regarded as perfect, professing as it does to describe only the general characteristics common to the whole, but to leave untouched those finer shades by which the Afghans, the Parsees, the Gentoos, and other classes, are discriminated from each other. The native inhabitants of Bengal are in general a handsome race. They possess in common with Europeans the Caucasian conformation of the head, the sides of which are in many individuals somewhat compressed. Their features are regular and well formed, with an expression of mildness. A great proportion of the natives of the lower provinces are of a small size and slight frame, yet many are found who, as well as their more favoured countrymen of the upper provinces, are of good stature and elegant proportion, and remarkable for grace and dignity of deportment. Some notice has already been taken in this article of their constitutional peculiarities, especially of their greater susceptibility of impression and slight capacity of resistance when compared with Europeans, as exemplified in the modification required in the treatment of the diseases which assail them. The author thinks, moreover, that the functions of the skin and conditions of the internal organs are not regulated by the same sympathies, and do not exert on each other the same mutual influence, as in Europeans. To illustrate this position he states that from twenty-five to thirty thousand bathe every morning in the river Hoogly at Calcutta and in its vicinity; they all wash their muslin clothing at the time of bathing, and, with very few exceptions, both men and women walk home in their wet clothes, some of them to a distance of two or three miles. Their religious customs require them to bathe more frequently in the month of November than at any other season, and the morning air is then usually cold; notwithstanding which, they never contract disease by an exposure in wet clothes under circumstances in which it would certainly produce illness in half of any given number of Europeans similarly situated. Yet these same people, who bear this chilling with impunity, sleep under the direct rays of the sun in the hot season, covered only by a common white muslin cloth, and when suffering from fever will not allow the contact of cold water even to wash their face and hands, but are heated by means of pans of burning charcoal to extract the cold which they think has entered the body and caused the disease! The author conjectures that the cause of this tolerance of vicissitudes is to be sought partly in the peculiar structure and functions of the skin and partly in habitual exposure. A good deal, we think, is to be ascribed to the principle that the exposure to high temperatures increases the calorific power of the body, and consequently that of resisting cold, during a period of considerable duration. Besides the experiments of Dr. Milne Edwards on the subject, this is familiarly exemplified in the case of glass-makers and other artisans in our own country, and is strongly illustrated by the fact mentioned by Count Ségur, that the intense cold of the Russian campaign of 1813 was better supported by Italians, Spaniards, and other natives of the south of Europe, than by those of the north. From the converse of this principle, the depression of the calorific power by cold, it is probable that the Hindoos find their frequent ablutions and lengthened wettings instrumental in mitigating the effects of the ardent heat of their climate.

The natives are liable to certain diseases unknown to Europeans. One of the most remarkable of these is the *Nakra*, or *Nasa*, which literally means the nose-disease. It appears to consist of a sudden and violent congestion of blood in the Schneiderian membrane, which extends to the mucous lining of the sinuses of the frontal, malar, maxillary and ethmoid bones, with pyrexia remarkable rather for rapidity of the circulation than for augmentation of its force. There is great pain in the parts which are the seat of the affection, and in the back of the neck and in the loins, and great heat of forehead. The duration of the disease is from three to five days. It never proves directly fatal, but is sometimes so by the intervention of *Biggar*, a very dangerous disease, with intense cerebral symptoms, to which it occasionally gives rise. The only remedy employed by the notives for *Nakra* is the drawing of blood, by thrusting a rough sharp-edged grass up the nostrils, from the Schneiderian membrane, or puncturing it with a sort of awl. In this way about an ounce of blood is obtained, which affords great relief. Mr. Twining supposes that this disease bears some analogy to one mentioned by Dr. Rush as prevalent in North Carolina, and known there under the name of pleurisy of the head.

That peculiar affection of the cellular membrane of which *Bucnemia Tropica* (GOOD) is a modification, though acknowledged to be very rare in Europeans, has been observed by Mr. Twining in one individual of the mixed Indo-Portuguese race, in whom, however, the Asiatic blood appeared to predominate. In this disease, the local affection, which consists of an infiltration of serum into the cellular membrane, is associated with irregular intermittent fever. In the case of the Indo-Portuguese, the infiltration took place into the cellular membrane of the left breast. There was a diffused swelling and a depressed cicatrix, which at the commencement of the hot stage of the third or fourth paroxysm opened spontaneously, and a quantity of limpid fluid flowed out. Twelve ounces are sometimes voided in this way in the space of an hour, the fluid in a short time coagulating firmly. The discharge is speedily followed by the cure of the fever, the patient remaining well for several months. A similar series of phenomena, general and local, accompanies that very common form of the disease which is seated in the scrotum. This form is very frequent in the upper provinces, and a very interesting account of it, as it prevails in the vicinity of Midnapore, is given by Dr. Goodeve, in the seventh volume of the Calcutta Transactions. The bucnemia tropica or Cochin leg is in general attended in its early stage with returns of fever once or twice a month; it is, however, in many respects different, both as regards the local affection and the fever, from those cases in which the fluid is evacuated. It is not confined to Asiatics.

We regret that the length to which this article has already extended deters us from presenting the reader with a more copious notice of the valuable matter contained in the Calcutta Transactions than we have yet done. It would, for instance, have been a gratification to us to analyze the paper of Mr. Bramley, in the sixth volume, on the Bronchocele of Nipâl and the Cis- and Trans-Himalayan regions, considering it at once the most learned and the most practical treatise on this disease that has fallen under our observation. The very interesting and extensive researches (conducted over a space of one thousand square miles) of Mr. M'Clelland on the connexion between the lime-stone formation and the same disease, contained in the seventh volume, contain results which, if confirmed by observations in other quarters of the world, will prove of the greatest importance, relatively to the etiology of *goitre*, and probably of some other affections. The papers of Dr. Wallich on some rare and curious plants of Bengal, in the same volume, contain matters of much interest to the botanist. In the sixth volume, there is a short article by the indefatigable author of the "Illustrations," deserving attention, because it refers to an important fact in the medical history of an agent, iodine, the properties of which are very generally undergoing investigation by the profession. He found that in five out of twenty-three cases of various diseases in which he employed this medicine, pain in the right side occurred, in some in the region of the gall-bladder, but more generally near the ninth rib. He hence infers that the greatest caution is requisite in prescribing it for patients in whom any affection of the liver exists. He regards these facts, moreover, as confirmatory of an opinion expressed by Professor Christison in his excellent work on poisons, that "iodine probably possesses the power of inflaming the liver,"—an opinion supported by two cases, the one resting on the authority of Rust, the other on that of Dr. Zinc, in which death, apparently caused by hepatitis, arose from the excessive employment of this medicine. Experience, however, varies greatly on this point: a case recently fell under our own observation in which the employment of iodine for chronic enlargement of the uterus was followed by acute pain in the hepatic region, extending thence to the right shoulder, and requiring the free application of leeches and mercurial purgatives for its removal. On the other hand, a chronic enlargement of the liver, to such an extent that the organ extended below the umbilicus, the sequel of remittent fever in a youth of seventeen, was entirely dispersed by frictions with a strong ointment of iodine and a course of purgatives.

In concluding this article, it is needless to express our opinion of Mr. Twining's work, we having sufficiently recorded it already. It will, henceforward, be the British practitioner's tropical guide. The materials of the "Transactions" are of course miscellaneous as to subjects, and in some degree so as to quality, but their general character in the latter respect entitles these volumes to a very high rank among publications of this description. We regret that we do not feel justified in speaking in terms of commendation of Mr. Conwell's book, and the more so as it contains evidence of industry, and of considerable, perhaps excessive, ingenuity.

His errors as a writer seem to arise, principally, from ignorance of the wants and taste of the medical reader. From this must have arisen his dedication of forty-two pages to a description of the nerves of the liver, which in a work of anatomy would have been deemed tedious, but in one of practical medicine is preposterous. To a cause of this kind must be ascribed, likewise, his three hundred pages (nearly) of abridged cases, a surfeit to the most voracious appetite for this sort of reading. We would suggest, too, that a style in reality diffuse, though wearing the outward aspect of conciseness and abruptness even to affectation, is but little calculated to win the fastidious ear of the public.

---

